# Addressing meniscal deficiency part 1: An umbrella review of systematic reviews and meta‐analyses on meniscal allograft transplantation

**DOI:** 10.1002/jeo2.12107

**Published:** 2024-09-30

**Authors:** Kevin A. Wu, Lulla V. Kiwinda, Aaron D. Therien, Christian J. Castillo, Stephanie Hendren, Jason S. Long, Annunziato Amendola, Brian C. Lau

**Affiliations:** ^1^ Department of Orthopaedic Surgery Duke University Durham North Carolina USA; ^2^ School of Osteopathic Medicine Campbell University Lillington North Carolina USA; ^3^ Medical Center Library & Archives Duke University School of Medicine Durham North Carolina USA

**Keywords:** meniscal allograft transplantation, meniscal injuries, osteoarthritis, scaffold‐based strategies, systematic review

## Abstract

**Purpose:**

Meniscal injuries are common in the young and active population. There is increasing utilization of surgical interventions like meniscal allograft transplantation (MAT) to restore the protective function of menisci following injury leading to meniscal deficiency. Extensive research and publications exist on the management of meniscal injury and the sequalae of meniscal deficiency. However, a comprehensive synthesis of the existing evidence through an umbrella review is lacking. This study aims to fill this gap by providing a current examination of the literature on MAT.

**Methods:**

A comprehensive search was conducted in the MEDLINE, Embase and Scopus databases to identify relevant systematic reviews and meta‐analyses. Studies were screened based on predefined inclusion and exclusion criteria. The quality of the included studies was assessed using the AMSTAR‐2 tool.

**Results:**

A total of 41 studies were included in the review, with most published within the last decade. The majority of studies (56.1%) received a ‘Critically Low’ confidence rating, 26.8% were rated as ‘Low’, and only 14.6% were rated as ‘High’ confidence. From the included studies, 51.2% reported on PROMs, with the Lysholm score being the most common. Transplant failure and reoperation rate were reported in 34.1% and 19.5% of studies respectively. Studies on MAT reported favourable short‐term outcomes in terms of patient‐reported outcome measures (PROMs) but were limited by the lack of randomized control trials and consistent comparison groups.

**Conclusions:**

This umbrella review highlights an increase in interest in MAT but underscores the need for higher‐quality reviews with standardized reporting and rigorous methodologies. Future research should focus on long‐term outcomes, optimal surgical techniques, patient selection criteria and risk factors for transplant failure. There is also a need for more studies focusing on MAT in pediatric populations. Overall, this review provides a comprehensive assessment of the current state of research in MAT and identifies areas for improvement in future studies.

**Level of Evidence:**

Level IV.

**Abbreviations**
ACI: autologous chondrocyte implantation
ACLR: anterior cruciate ligament reconstruction
AJSM: American Journal of Sports Medicine
AMSTAR‐2: a measurement tool to assess systematic reviews 2
CI: confidence interval
ESSKA: European Society of Sports Traumatology, Knee Surgery, Arthroscopy
HTO: high tibial osteotomy
IKDC: International Knee Documentation Committee
KOOS: Knee Injury and Osteoarthritis Outcome Score
KSSTA: knee surgery, sports traumatology, arthroscopy
MAT: meniscal allograft transplantation
OJSM: Orthopaedic Journal of Sports Medicine
OR: odds ratio
PRISMA: preferred reporting items for systematic reviews and meta‐analyses
PROM: patient reported outcome measures
RoB: risk of bias
RTS: return to sport
VAS: Visual Analogue Scale

## INTRODUCTION

Meniscal injuries pose a significant challenge in orthopedic surgery with an incidence of tears between 60 and 70 per 100,000 [5, 26]. The meniscus plays a crucial role in joint stability, load distribution and overall functionality [5, 33]. Treatment for meniscal injuries includes nonsurgical approaches like physical therapy, as well as surgical interventions such as meniscus repair, meniscectomy and meniscal allograft transplantation (MAT) [35]. In cases where preservation is not an option, MAT may be considered to help distribute the load more evenly compared to total meniscectomy; however, there has been limited evidence demonstrating that MAT delays the onset of osteoarthritis [15]. Although still a relatively uncommon procedure, the utilization of MAT is on the rise, with the incidence increasing from 0.12 to 0.15 per 100,000 between 2010 and 2019 in the United States [2, 12]. Kazi and colleagues demonstrated somewhat promising outcomes in a study of 86 MATs [25]. Although 71% of the transplants remained functional, 28% degenerated and required eventual total knee arthroplasty at an average follow‐up of 15 years. As a result, long term generalizable outcomes and patient selection are still being studied.

Suitable candidates for MAT are usually individuals in the young to middle‐aged range who are experiencing moderate to severe pain after meniscectomy [59]. There are various types of allografts available for meniscal allograft transplantation (MAT), including fresh‐frozen and irradiated or nonirradiated grafts. Additionally, different techniques can be employed, such as using or not using bone plugs, or incorporating a bone bridge between the roots [24, 45]. Alternatively, scaffold‐based strategies involve the use of synthetic or biological materials to create a supportive framework within the knee joint and may be performed in cases of partial meniscectomy [17]. The scaffold encourages tissue regeneration and repair, aiming to provide structural support akin to a natural meniscus [60]. Scaffold approaches are often considered for cases where transplantation may not be feasible or as a potential alternative to preserve meniscal function [17]. The decision between transplantation and scaffold depends on various factors, including the extent of meniscal damage, patient age, activity level and overall joint health [13]. This prompts a deeper exploration of what remains unknown in the landscape of meniscal transplantation, urging a critical examination of the existing literature to identify gaps in knowledge.

Given the growing interest in MAT and the extensive research on scaffold use, conducting an umbrella review to summarize the published evidence is necessary. Existing systematic reviews and studies on this topic may be outdated, incomplete or fail to address certain areas of interest. This is part one of a two‐part review article addressing controversial questions in meniscal interventions for deficiency [64]. Part One focuses on MAT while Part Two focuses on scaffold‐based interventions. The objectives of this study and umbrella review are threefold: (1) to conduct a systematic review of existing systematic reviews and meta‐analyses concerning MAT; (2) to assess the quality, merits and drawbacks of the published evidence found in peer‐reviewed literature; and (3) to pinpoint areas of deficiency in current research, thereby delineating avenues for future investigation. This umbrella review seeks to conduct a systematic review of existing systematic reviews and meta‐analyses concerning MAT, assess the quality of published evidence, and pinpoint areas of deficiency in current research.

## METHODS

A comprehensive umbrella review of systematic reviews and meta‐analyses was carried out following the guidelines of preferred reporting items for systematic reviews and meta‐analysis (PRISMA) [10, 36, 43].

### Search strategy

The search was conducted in the MEDLINE (Pubmed), Embase (Elsevier) and Scopus (Elsevier) databases to identify relevant publications. The search encompassed results from the inception of these databases until 9 September 2021, with a subsequent updated search conducted on 23 October 2023. A medical librarian (SH) performed searches using key terms such as ‘meniscus’, ‘scaffold/transplant’, and ‘systematic review’ employing a Boolean search strategy (Table S1). After duplicated studies were removed, two independent reviewers (L. K., A. T.) manually screened the titles and abstracts of all the studies by employing predetermined exclusion criteria. Afterwards, a predetermined inclusion criteria was used by both reviewers to evaluate the full texts of potential studies. Disagreements about study inclusion were resolved by discussion between the two independent reviewers and any remaining disagreements were solved by a third reviewer (K. W.). For example, in one instance, there was initial disagreement regarding the inclusion of a study due to ambiguity in the reporting of MAT outcomes. This was resolved by referring back to the inclusion criteria and consulting a third reviewer, resulting in the exclusion of the study.

### Inclusion and exclusion criteria

Predetermined inclusion and exclusion criteria were agreed upon by all authors before initiating the study. Inclusion criteria constituted studies that: (1) contained relevant data concerning MAT, (2) were published in the English language, (3) were published in peer review journals, (4) were systematic reviews or/and meta‐analysis and (5) reported outcomes related to MAT, such as patient‐reported outcomes, radiographic assessments or surgical techniques. Studies were excluded if they: (1) did not contain relevant outcomes about MAT, (2) were abstract only studies and (3) were written in a language other than English.

A template for data extraction was developed, covering citation details, study purpose, surgical technique and main study outcomes for each included study.

### Quality of the studies

The quality of the articles included in this study was assessed using the AMSTAR‐2 tool, which consists of 16 criteria [49]. Two researchers (L. K., A. T.) independently analyzed the quality of each eligible article. In cases of disagreement, consensus was reached through discussion or with a third reviewer (K. W.).

Confidence in the results of each systematic review was determined by the AMSTAR‐2 tool and categorized as follows: (1) ‘High’—zero or one noncritical weakness: the systematic review provides a comprehensive and accurate summary of results; (2) ‘Moderate’—more than one noncritical weakness but no critical flaws: the review gives an accurate summary of the results; (3) ‘Low’—one critical flaw, with or without noncritical weaknesses: the review may not give a comprehensive or accurate review of the results; or (4) ‘Critically low’—more than one critical flaw, with or without noncritical weaknesses: the systematic review should not be used to provide a summary of the results.

### Statistical analysis

Quantitative statistical analysis was performed in R (version 3.1; The R Foundation) to get frequencies.

## RESULTS

**Figure 1:**
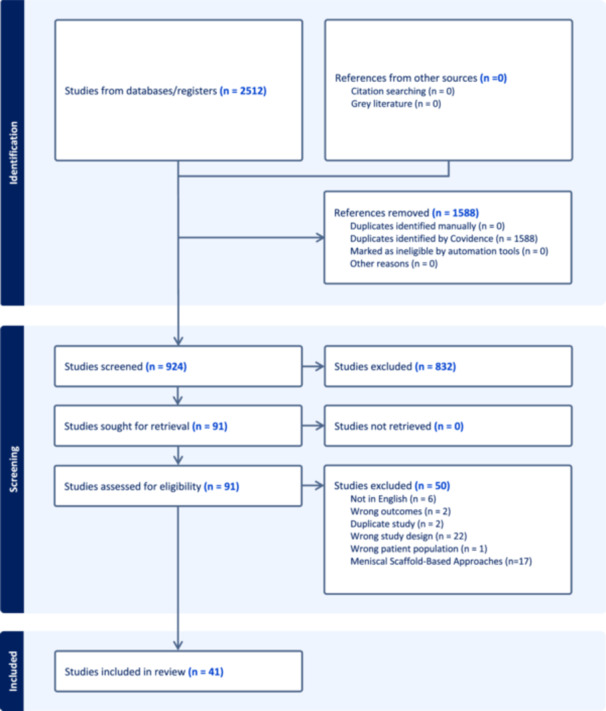
The preferred reporting items for systematic reviews and meta‐analyses (PRISMA) flowchart illustrates the screening process and selection of final articles for reviews focusing on meniscal allograft transplantation approaches.

A total of 2512 studies were identified in the database searches and exported a reference manager software (EndNoteTM X9; Clarivate Analytics). The references were uploaded onto a systematic review screening software (Covidence). Any duplicates (1588 references) were eliminated, and the remaining 924 were screened based on their title and abstract for relevance. An additional 832 studies were determined to be irrelevant, and the full text of 91 studies were examined in detail. During the full text review, 32 articles were rejected because they did not meet the appropriate inclusion and exclusion criteria. There were 58 studies that focused on meniscal interventions. There were 41 reviews focusing on MAT and were included in this umbrella review (Figure 1) [1, 6, 7, 8, 11, 13, 14, 15, 18, 19, 21, 23, 29, 30, 31, 34, 37, 38, 39, 40, 41, 44, 45, 46, 48, 51, 52, 62, 63].

The most common reason for the exclusion of full text was study design (*n* = 22). There were 17 studies that were excluded for focusing on meniscal scaffold‐based approaches and were included in the accompanying article (part 2) [64]. Most of the studies were published recently with 87.8% (36/41) of the studies published within the last 10 years (i.e., 2013–2023) and 24.4% (10/41) of the studies published within the last two years (i.e., 2021–2023). The oldest systematic review was published in 2007. There were 10 meta‐analyses (24.4%) and 31 systematic reviews (75.6%) included within the study.

### Quality of the studies

The overall methodological quality of the 41 studies included within the review is summarized in Table 1. Using the AMSTAR‐2 criteria, the majority of studies (*n* = 23; 56.1%) received an overall confidence rating of ‘Critically Low’ [49]. Additionally, 11 reviews (26.8%) were rated as ‘Low’ confidence, while 1 review (2.4%) received a ‘Moderate’ rating. Only 6 (14.6%) of the included reviews were rated as ‘High’ confidence. These findings highlight a pervasive lack of confidence in the results of most systematic and meta‐analysis reviews on MAT, as per the AMSTAR‐2 quality rating criteria. These results mirror those observed in other areas of orthopedic research [3, 47, 64].

**Table 1:** AMSTAR 2 assessment of the included studies.

	AMSTAR 2—Items																
References	1	2	3	4	5	6	7	8	9	10	11	12	13	14	15	16	Overall items
Leite et al. [32]	Yes	Yes	Yes	Yes	Yes	Yes	Yes	Yes	Yes	Yes	No MA	No MA	No	Yes	Yes	Yes	Low
Kunze et al. [28]	Yes	Yes	Yes	Yes	No	No	No	Yes	Yes	Yes	Yes	Yes	Yes	Yes	Yes	Yes	Low
Turati et al. [57]	Yes	Yes	Yes	Yes	Yes	Yes	No	Yes	Yes	Yes	No MA	No MA	Yes	Yes	No MA	Yes	Low
Candura et al. [9]	Yes	Yes	Yes	Yes	Yes	Yes	Yes	Yes	No	Yes	No MA	No MA	Yes	Yes	No	No	Critically low
Tan et al. [55]	Yes	Yes	Yes	Yes	Yes	Yes	Yes	Yes	Yes	Yes	No MA	No MA	Yes	Yes	Yes	No	High
Ow et al. [42]	Yes	Yes	Yes	Yes	Yes	Yes	Yes	Yes	Yes	No	Yes	Yes	Yes	Yes	Yes	Yes	High
Knapik et al. [27]	Yes	Yes	Yes	Yes	No	No	No	Yes	No	Yes	No MA	No MA	No	No	No	Yes	Critically low
Wang et al. [61]	Yes	No	Yes	Yes	Yes	Yes	No	Yes	Yes	Yes	No MA	No MA	Yes	Yes	No MA	Yes	Critically low
Su et al. [54]	Yes	Yes	Yes	Yes	Yes	No	No	Yes	No	Yes	No MA	No MA	No	Yes	No MA	Yes	Critically low
Fanelli et al. [16]	Yes	Yes	Yes	Yes	Yes	Yes	Yes	Yes	Yes	Yes	Yes	Yes	Yes	Yes	Yes	Yes	High
Smoak et al. [53]	Yes	Yes	Yes	Yes	Yes	Yes	No	Yes	Yes	Yes	No MA	PY	Yes	Yes	No MA	Yes	Low
Hurley et al. [22]	Yes	Yes	Yes	Yes	Yes	Yes	Yes	Yes	No	Yes	No MA	No MA	Yes	No	No	Yes	Critically low
Waugh et al. [62]	Yes	Yes	Yes	Yes	Yes	Yes	Yes	Yes	Yes	Yes	No MA	No MA	Yes	Yes	No MA	Yes	High
Seitz et al. [48]	Yes	Yes	Yes	Yes	Yes	Yes	No	Yes	No	PY	No MA	No MA	No	No	No MA	Yes	Critically low
Novaretti et al. [39]	Yes	Yes	Yes	Yes	Yes	Yes	No	Yes	Yes	PY	No MA	No MA	Yes	Yes	No MA	Yes	Low
Lee et al. [31]	No	Yes	Yes	Yes	No	No	No	Yes	Yes	Yes	No MA	No MA	Yes	No	No	Yes	Critically low
Grassi et al. [18]	Yes	Yes	Yes	Yes	Yes	Yes	Yes	Yes	Yes	PY	Yes	No	No	Yes	Yes	Yes	Low
Lee et al. [30]	Yes	Yes	Yes	Yes	Yes	Yes	Yes	Yes	Yes	No	Yes	Yes	Yes	Yes	Yes	Yes	High
Lee et al. [29]	Yes	Yes	Yes	Yes	Yes	Yes	No	Yes	Yes	No	Yes	Yes	Yes	Yes	No	Yes	Critically low
Jauregui et al. [23]	Yes	Yes	Yes	Yes	No	No	No	Yes	No	No	Yes	No	Yes	No	No	Yes	Critically low
Bin et al. [8]	Yes	Yes	Yes	Yes	Yes	Yes	Yes	Yes	No	No	Yes	Yes	Yes	Yes	No	No	Critically low
Bin et al. [7]	Yes	Yes	Yes	Yes	Yes	Yes	Yes	Yes	No	No	Yes	Yes	Yes	Yes	No	No	Critically low
Barber‐Westin et al. [6]	Yes	Yes	Yes	Yes	No	No	Yes	Yes	No	Yes	No MA	No MA	No	No	No MA	Yes	Critically low
Afzali et al. [1]	Yes	Yes	Yes	Yes	Yes	Yes	Yes	Yes	Yes	Yes	No MA	No MA	Yes	Yes	No MA	Yes	High
Monk et al. [37]	Yes	Yes	Yes	Yes	Yes	Yes	No	Yes	Yes	Yes	No MA	No MA	Yes	Yes	No MA	Yes	Low
DeBruycker et al. [14]	Yes	Yes	Yes	Yes	No	No	Yes	Yes	No	PY	Yes	No	No	Yes	No	No	Critically low
Dangelmajer et al. [13]	Yes	Yes	Yes	Yes	Yes	Yes	Yes	Yes	No	PY	No MA	No MA	Yes	Yes	No	No	Critically low
Wei et al. [63]	Yes	No	Yes	Yes	No	Yes	No	Yes	Yes	Yes	Yes	Yes	Yes	Yes	PY	Yes	Critically low
Smith et al. [52]	Yes	Yes	Yes	Yes	Yes	Yes	No	Yes	Yes	No	No MA	No MA	Yes	Yes	No MA	Yes	Low
Smith et al. [51]	Yes	Yes	Yes	Yes	No	No	No	Yes	Yes	No	No MA	No MA	No	No	No MA	Yes	Critically low
Samitier et al. [46]	Yes	Yes	Yes	Yes	No	Yes	No	Yes	Yes	No	No MA	No MA	Yes	No	No MA	No	Low
Samitier et al. [45]	Yes	Yes	Yes	Yes	No	Yes	No	Yes	Yes	No	No MA	No MA	Yes	Yes	No MA	No	Low
Rosso et al. [44]	Yes	No	Yes	Yes	Yes	Yes	No	Yes	Yes	Yes	No MA	No MA	No	Yes	No MA	Yes	Critically low
Oh et al. [41]	Yes	No	Yes	Yes	No	No	No	Yes	No	Yes	No MA	No MA	No	Yes	No MA	Yes	Critically low
Noyes et al. [40]	Yes	No	Yes	Yes	No	No	No	Yes	No	No	No MA	No MA	No	No	No MA	No	Critically low
Myers et al. [38]	Yes	Yes	Yes	Yes	No	Yes	No	Yes	Yes	PY	No MA	No MA	No	Yes	No MA	Yes	Critically low
Harris et al. [19]	Yes	Yes	Yes	Yes	Yes	No	Yes	Yes	Yes	No	No MA	No MA	Yes	Yes	Yes	Yes	Moderate
Elattar et al. [15]	Yes	Yes	Yes	Yes	No	No	No	Yes	Yes	No	Yes	No	Yes	Yes	No	No	Critically low
Hergan et al. [21]	Yes	Yes	Yes	Yes	Yes	Yes	Yes	Yes	No	No	No MA	No MA	Yes	Yes	Yes	Yes	Low
Crook et al. [11]	Yes	No	No	No	No	No	No	Yes	No	No	No MA	No MA	No	No	No MA	Yes	Critically low
Matava et al. [34]	Yes	No	Yes	Yes	No	No	No	Yes	No	No	No MA	No MA	No	No	No MA	Yes	Critically low

*Note*: Description of AMSTAR‐2 Items: 1—Did the research questions and inclusion criteria for the review include the components of PICO?; 2—Did the report of the review contain an explicit statement that the review methods were established prior to the conduct of the review and did the report justify any significant deviations from the protocol?; 3—Did the review authors explain their selection of the study designs for inclusion in the review?; 4—Did the review authors use a comprehensive literature search strategy?; 5—Did the review authors perform study selection in duplicate?; 6—Did the review authors perform data extraction in duplicate?; 7—Did the review authors provide a list of excluded studies and justify the exclusions?; 8—Did the review authors describe the included studies in adequate detail?; 9—Did the review authors use a satisfactory technique for assessing the risk of bias (RoB) in individual studies that were included in the review?; 10—Did the review authors report on the sources of funding for the studies included in the review?; 11—If meta‐analysis was performed did the review authors use appropriate methods for statistical combination of results?; 12—If meta‐analysis was performed, did the review authors assess the potential impact of RoB in individual studies on the results of the meta‐analysis or other evidence synthesis?; 13—Did the review authors account for RoB in individual studies when interpreting/discussing the results of the review?; and 14—Did the review authors provide a satisfactory explanation for, and discussion of, any heterogeneity observed in the results of the review?; 15—If they performed quantitative synthesis did the review authors carry out an adequate investigation of publication bias (small study bias) and discuss its likely impact on the results of the review?; 16—Did the review authors report any potential sources of conflict of interest, including any funding they received for conducting the review?

Abbreviations: No MA, no meta‐analysis; PY, partial yes.

We did not exclude any articles from further analysis due to the quality assessment as this study would allow for investigators to understand areas in which to improve the quality of systematic reviews related to MAT. Additionally, including all these studies would enable a comprehensive review and assessment of the existing literature base.

The majority of studies included in the review did not include a protocol registration in a register such as PROSPERO. There were only 17 studies (41.4%) that provided a list of excluded studies and justified the exclusions. Approximately 39.0% of the studies did not provide an adequate technique for assessing the risk of bias (RoB) within studies. Furthermore, studies commonly did not account for RoB during their discussion and interpretation of the results (39.0%).

### Studies characteristics

The 41 reviews were published in 18 distinct journals. The top four journals where these studies were published include the Knee Surgery, Sports Traumatology, Arthroscopy (KSSTA) (24.4%), American Journal of Sports Medicine (AJSM) (17.1%), Arthroscopy (14.6%) and Orthopaedic Journal of Sports Medicine (OJSM) (7.3%) (Table 2). The top five countries where the studies were conducted were the United States (36.6%), South Korea (14.6%), United Kingdom (14.6%) and Italy (12.2%). On average, these reviews included 21.3 studies with most of them being retrospective cohort studies (Table 3). The majority of reviews included studies that were predominantly made up of male participants ranging from 57%–81% [6, 9, 14, 19, 22, 23, 34, 39, 40, 42, 52, 62, 63]. One review, focusing on MAT in skeletally immature patients, included studies predominantly involving females with a limited sample size of 58 patients [57].

**Table 2:** The most common journals and countries of origin.

	Studies (*n*)	Studies (%)
*Journal*		
Knee Surgery, Sports Traumatology, Arthroscopy	10	24.4
The American Journal of Sports Medicine	7	17.1
Arthroscopy: The Journal of Arthroscopic and Related Surgery	6	14.6
The Orthopaedic Journal of Sports Medicine	3	7.3
*Country of origin*		
United States	15	36.6
South Korea	6	14.6
United Kingdom	6	14.6
Italy	5	12.2

**Table 3:** Demographics of the included studies.

Study	Aim of study	Number of studies/participants	Age of participants, range/mean	Percentage male
Leite et al. [32]	To systematically summarize the medial meniscus allograft transplantation (MAT) reported outcomes and evaluate whether the surgical technique is associated with allograft extrusion and knee function	328 patients	Range: 17–53 years	N/A
Kunze et al. [28]	To perform a systematic review and meta‐analysis of risk factors associated with graft failure after MAT of the knee	2184 patients	35.1 ± 5.2 years	N/A
Turati et al. [57]	To perform a systematic review of MAT in skeletally immature patients to better understand which patients can benefit most from this surgery and to describe postoperative clinical outcomes regarding knee pain, function and the need for reoperation	3 studies, 58 patients	N/A	33%
Candura et al. [9]	To evaluate the clinical and functional outcomes of meniscal allograft transplantation (MAT) with anterior cruciate ligament reconstruction (ACLR)	138 (135 patients)	35.3 years	69.20%
Tan et al. [55]	To evaluate the clinical outcomes of anterior cruciate ligament reconstruction (ACLR) with meniscal allograft transplantation (MAT) through a systematic review of current available evidence	363 patients	30.5 years	N/A
Ow et al. [42]	To perform a systematic review and meta‐analysis to compare the clinical and radiological outcomes of various methods of allograft fixation in MAT	2604 patients	34.1 years	66.30%
Knapik et al. [27]	To review the literature on RTS rates, predictors of RTS success or failure, and patient‐reported outcomes following MAT in athletic patients	297 patients	N/A	N/A
Wang et al. [61]	To understand prognostic factors for survivorship using evidence‐based selection criteria in order to identify patients who would best benefit from meniscal allograft transplant (MAT)	18 studies, 1920 patients	N/A	N/A
Su et al. [54]	The purpose of this study was to perform a systematic review of clinical outcome studies of patients undergoing MAT and to compare postoperative improvements with MCID thresholds	35 studies, 1658 patients	N/A	N/A
Fanelli et al. [16]	Investigate variables that can predict patient‐reported outcome measures (PROMs) and failure of MAT in symptomatic postmeniscectomy patients	3460 (3382 patients)	34 ± 11 years	N/A
Smoak et al. [53]	To provide a qualitative summary of the published systematic reviews and meta‐analyses regarding the meniscus	142 studies	Adults aged 40 and older	N/A
Hurley et al. [22]	To systematically review the literature and to evaluate the reported rehabilitation protocols, return‐to‐play guidelines and subsequent rates of return‐to‐play following MAT	3321 patients	33 years	68.56%
Waugh et al. [62]	To assess the clinical effectiveness and cost‐effectiveness of meniscal allograft transplantation (MAT) after meniscal injury and subsequent meniscectomy	1731 patients	25–45 years	48 to 80%
Seitz and Dürselen [48]	To provide an overview of the current knowledge on how allografts used for the reconstruction of ligaments and the meniscus are integrated and perform specifically regarding their biomechanical function	19 studies of MAT, 1731 patients	N/A	N/A
Novaretti et al. [39]	The purpose of this systematic review was to investigate the long‐term survivorship rates and functional outcomes of MAT in patients with minimum 10‐year postoperative follow‐up	11 studies, 688 (658 patients)	33.1 years	63%
Lee et al. [31]	To determine the time to and rate of the return to sports (RTS) after meniscal surgery and to compare these values among the different types of meniscal surgeries	514 patients	25.1 ± 6.2 years	N/A
Grassi et al. [18]	To evaluate return to sport, clinical outcome, and complications after MAT in sport‐active patients	467 patients	31.1 ± 6.2 years	N/A
Lee [30]	To quantify the incidence and extent of graft extrusion after MAT	21 studies	N/A	N/A
Lee et al. [29]	To evaluate whether there is a difference in clinical outcomes between isolated MAT and MAT combined with other procedures (combined MAT)	1882 patients	Range: 19.8–45.3 years	N/A
Jauregui et al. [23]	To assess the overall outcome of MAT and compare the results of different meniscal root fixation techniques	1637 patients	33.5 years	81%
Bin et al. [8]	To assess whether lateral meniscal allograft transplantation (MAT) procedures lead to better clinical outcomes than medial MAT	287 medial MAT, 407 lateral MAT	N/A	N/A
Bin et al. [7]	To compare the incidence and amount of transplanted meniscus allograft extrusion following medial and lateral MAT	481 patients	N/A	N/A
Barber‐Westin and Noyes [6]	To determine sports activities achieved after meniscus transplantation and if associations exist between sports activity levels and transplant failure or progression of tibiofemoral osteoarthritis (OA)	1497 patients (1521 menisci)	34.3 ± 6.7 year	70.36%
Afzali et al. [1]	To gather and appraise the cost‐effectiveness of treatment approaches for nonosteoarthritic knee pain conditions	15 studies	N/A	N/A
Monk et al. [37]	To compare the effectiveness of arthroscopic surgery for meniscal injuries in all populations	4904 patients	N/A	N/A
DeBruycker et al. [14]	To evaluate the mid‐ to long‐term survival outcome of MAT (meniscal allograft transplantation)	2977 patients (3157 allografts)	33 years	66.58%
Dangelmajer et al. [13]	To compare the success and failure rates of two techniques, MAT and meniscal scaffolds, and make an inference which treatment is more preferable at the present time and future	1197 patients	N/A	N/A
Wei et al. [63]	To perform a literature review and meta‐analysis evaluating the effectiveness of medial and lateral meniscus allograft transplantation (MAT)	12 studies, 676 patients	Range: 31–40 years	66%
Smith et al. [52]	The purpose of this systematic review was to examine the hypothesis that meniscal allograft transplantation is chondroprotective by identifying and appraising studies that have assessed the progression of OA following meniscal allograft transplantation	38 studies, 1056 MAT	Range: 15–50 years	N/A
Smith et al. [51]	The primary objective of this study was to perform an updated systematic review and meta‐analysis of meniscal allograft transplantation using patient reported outcome measures at final follow‐up as the outcome tool. The secondary objective was to provide an up to date review of the indications, associated procedures, operative technique, rehabilitation, failures, complications, radiological outcomes and graft healing.	35 studies, 1332 patients	33.7 years	57%
Samitier et al. [46]	To provide a systematic review of the literature regarding five topics in meniscal allograft transplantation (MAT): Optimal timing for transplantation, outcomes, return to competition, associated procedures and prevention of osteoarthritis	24 studies	N/A	N/A
Samitier et al. [45]	The purpose of this study was to provide a comprehensive and updated systematic review of the literature regarding five controversial issues: Graft biology, shrinkage, extrusion, sizing and fixation	62 studies	N/A	N/A
Rosso et al. [44]	The aims of this study were to assess (1) the quality of the published studies on MAT; (2) the indications for this type of surgery; (3) the methods used for preservation, sizing, and fixation of the allograft; and (4) the clinical and radiographic outcomes of this procedure and its role in preventing osteoarthritis	55 studies	Range 19.3–46.9	N/A
Oh et al. [41]	The purpose of this review was to assess the need and to ascertain the indication and the role of second‐look arthroscopy for objective evaluation after MAT	15 studies, 541 patients	N/A	N/A
Noyes and Barber‐Westin [40]	The purpose of this study is to determine the incidence of meniscus transplant extrusion, the effect of graft fixation techniques on extrusion rates, and the clinical significance of this potential problem	23 studies, 1006 patients	34 years	70.8%
Myers and Tudor [38]	The purpose of this systematic review was to assess a number of the technical aspects of MAT that have not been covered in other reviews. Specific variables studied include the indications for MAT, graft fixation method, rehabilitation protocols, outcome scores and definition of failure	41 studies, 1849 surgeries	33.7 years	N/A
Harris et al. [19]	To compare outcomes after combined MAT and cartilage repair/restoration with the outcomes of isolated MAT or cartilage repair/restoration	110 patients	38.8 years	76.37%
Elattar et al. [15]	To present a meta‐analysis of published trials reporting outcomes of meniscal allograft transplantation to establish its safety and reproducibility	1068 patients (1136 grafts)	34.8 years	N/A
Hergan et al. [21]	To perform a systematic review of available literature to answer the following: (1) Does MAT prevent advancing chondrosis? (2) Who is the ideal candidate for MAT? (3) What is the survival time for allograft in a stable knee? (4) Can MAT be successful when performed with concomitant procedures? (5) Is there an outcome difference between medial and lateral meniscal allograft transplants? (6) What is the expected function of a knee that has undergone MAT?	323 patients	33.9 years	N/A
Crook et al. [11]	To review the current literature to consolidate the evidence surrounding the use of human meniscal allograft transplantation	N/A	N/A	N/A
Matava [34]	To address four questions: Who is the ideal candidate to undergo meniscal transplantation? What is the ideal method of graft sizing, preservation, and implantation? What is the accepted means of postoperative rehabilitation and timing of a return to athletic activities? And what is the overall success rate of this procedure based on validated knee‐outcome instruments?	15 studies, 516 patients	33.4 years (range, 14–55 years)	68%

### Meniscal allograft transplantation

The 41 studies that examined the use of MAT as a surgical intervention focused on multiple types of MATs, including those that included fresh‐frozen and cryopreserved allografts. These findings collectively suggest that while MAT can offer significant benefits, patient selection and addressing associated cartilage damage are crucial for long‐term success. Concomitant procedures, as reported by several studies, are common during MAT and include ACLR, HTO and microfractures.

Four studies examined potential risk factors associated with MAT failures and patient reported outcome measures (PROM) [16, 27, 28, 61]. Kunze and colleagues found that the pooled 5‐year failure rate was 10.9% (range, 4.7%–23%), and for 10‐year failure rates, it was 22.7% (range, 8.1%–55.0%) across studies for MAT [28]. They identified that an International Cartilage Regeneration & Joint Preservation Society grade >3a was a significant risk factor for failure after MAT (OR, 5.32; 95% CI, 2.75–10.31; *p* < 0.001), while there was no significant evidence for patient sex or MAT laterality as risk factors for failure after MAT. Wang and colleagues similarly found that severe cartilage damage was strongly associated with poor survivorship. Their study concluded that factors such as sex, knee compartment, surgical side, concomitant anterior cruciate ligament reconstruction (ACLR) and concomitant osteotomy for malalignment showed no significant effect while there was moderate evidence suggesting that body mass index (<36), tobacco use and arthroscopic versus open procedures did not influence survivorship [61]. Fanelli and colleagues demonstrated evidence that a higher number of previous procedures in the same knee predicted transplant failure [16]. They ultimately concluded that the ideal candidate for MAT may be a young male of normal weight with no previous knee surgeries, treated with a lateral isolated procedure.

Twenty‐one of the studies (51.2%) specified having a concomitant procedure done while four reviews (9.8%) included studies that were isolated to MAT. The three most common concomitant procedures performed were ACLR, high tibial osteotomy (HTO) and microfractures. Less commonly performed procedures were autologous chondrocyte implantation (ACI) and cartilage repairs [21, 50, 59].

There were 21 studies (51.2%) that reported on PROMs (Table 4) [8, 9, 14, 15, 16, 18, 19, 21, 23, 29, 32, 34, 38, 39, 40, 42, 44, 51, 54, 55, 57, 62, 63]. The most commonly reported PROMs were the Lysholm score (*n* = 21; 51.2%) followed by the International Knee Documentation Committee (IKDC) (*n* = 19; 46.3%), Tegner (*n* = 16; 39.0%), Visual Analogue Scale (VAS) (*n* = 12; 29.3%), Knee injury and Osteoarthritis Outcome Score (KOOS) (*n* = 7; 17.1%). All reviews that reported on PROMs concluded postoperative improvements compared to baseline. However, there was a noted high prevalence of concomitant procedures included in these studies. Transplant failure rate was reported within 14 studies (34.1%) while reoperation rate was reported in 8 studies (19.5%) [6, 9, 13, 14, 15, 16, 18, 19, 21, 23, 28, 32, 42, 63].

**Table 4:** Patient reported outcome measures, failure rate and reoperation rate following meniscal allograft transplantation.

Study	Intervention description	Base line PROMs	Postoperative PROMs	Mean follow‐up	Survivorship	Failure and reoperation rate
Leite et al. [32]	Medial MAT	VAS average: 5.2 VAS number of studies: 3 IKDC: 52.8 IKDC number of studies: 3 KOOS average: N/A KOOS number of studies: N/A Tegner average: 3.3 Tegner number of studies: 2 Lysholm average: 59.9 Lysholm number of studies: 7	VAS average: 2.9 VAS number of studies: 4 IKDC: 72.5 IKDC number of studies: 4 KOOS average: N/A KOOS number of studies: N/A Tegner average: 5.5 Tegner number of studies: 5 Lysholm average: 83 Lysholm number of studies: 9	(24–167) months	Survivorship 1: Percentage: 96.2% Follow‐up: 5 year Survivorship 2: Percentage: 88.8% Follow‐up: 10 year	Failure rate: 2.6% Number of studies: 2 Reoperation rate: 24.4% Number of studies: 4
Kunze et al. [28]	MAT+ACLR	VAS average: N/A VAS number of studies: N/A IKDC: N/A IKDC number of studies: N/A KOOS average: N/A KOOS number of studies: N/A Tegner average: N/A Tegner number of studies: N/A Lysholm average: N/A Lysholm number of studies: N/A	VAS average: N/A VAS number of studies: N/A IKDC: N/A IKDC number of studies: N/A KOOS average: N/A KOOS number of studies: N/A Tegner average: N/A Tegner number of studies: N/A Lysholm average: N/A Lysholm number of studies: N/A			Failure rate: 17.8% Number of studies: 17
Turati et al. [57]	MAT	VAS average: N/A VAS number of studies: N/A IKDC: 40.4 IKDC number of studies: 2 KOOS average: 67.1 KOOS number of studies: 2 Tegner average: N/A Tegner number of studies: N/A Lysholm average: 50.55 Lysholm number of studies: 2	VAS average: N/A VAS number of studies: N/A IKDC: 73.17 IKDC number of studies: 3 KOOS average: 80.79 KOOS number of studies: 2 Tegner average: 7 Tegner number of studies: 1 Lysholm average: 69.57 Lysholm number of studies: 3	54.3 months		
Candura et al. [9]	MAT+ACLR	VAS average: N/A VAS number of studies: N/A IKDC: 41.2 IKDC number of studies: 3 KOOS average: N/A KOOS number of studies: N/A Tegner average: 2.8 Tegner number of studies: 1 Lysholm average: 51.8 Lysholm number of studies: 3	VAS average: N/A VAS number of studies: N/A IKDC: 65.9 IKDC number of studies: 3 KOOS average: N/A KOOS number of studies: N/A Tegner average: 4.6 Tegner number of studies: 2 Lysholm average: 78.5 Lysholm number of studies: 5	5.3 years		Failure rate: 10.1% Number of studies: 4 Reoperation rate: 23.90% Number of studies: 4
Tan et al. [55]	MAT‐ACLR	VAS average: N/A VAS number of studies: N/A IKDC: 54.1 IKDC number of studies: 3 KOOS average: N/A KOOS number of studies: N/A Tegner average: 3.5 Tegner number of studies: 3 Lysholm average: 55.6 Lysholm number of studies: 5	VAS average: N/A VAS number of studies: N/A IKDC: 71.1 IKDC number of studies: 3 KOOS average: N/A KOOS number of studies: N/A Tegner average: 5.2 Tegner number of studies: 3 Lysholm average: 77.6 Lysholm number of studies: 5	4.08 years		
Ow et al. [42]	Bone plug MAT	VAS average: N/A VAS number of studies: N/A IKDC: N/A IKDC number of studies: N/A KOOS average: N/A KOOS number of studies: N/A Tegner average: N/A Tegner number of studies: N/A Lysholm average: N/A Lysholm number of studies: N/A	VAS average: N/A VAS number of studies: N/A IKDC: N/A IKDC number of studies: N/A KOOS average: N/A KOOS number of studies: N/A Tegner average: N/A Tegner number of studies: N/A Lysholm average: N/A Lysholm number of studies: N/A	4.8 (3.1–6.5) years		Failure rate: 6.2% (95% CI, 3.2%–11.6%) Number of studies: 8 Reoperation rate: 5.1% (95% CI, 1.9%–13.3%) Number of studies: 5
Wang et al. [61]	MAT	VAS average: N/A VAS number of studies: N/A IKDC: N/A IKDC number of studies: N/A KOOS average: N/A KOOS number of studies: N/A Tegner average: N/A Tegner number of studies: N/A Lysholm average: N/A Lysholm number of studies: N/A	VAS average: N/A VAS number of studies: N/A IKDC: N/A IKDC number of studies: N/A KOOS average: N/A KOOS number of studies: N/A Tegner average: N/A Tegner number of studies: N/A Lysholm average: N/A Lysholm number of studies: N/A	6.0 years	Survivorship 1: Percentage: 75%–96% Follow‐up: 5 year Survivorship 2: Percentage: 45%–89.4% Follow‐up: 10 year	
Su et al. [54]	MAT	VAS average: 5.92 VAS number of studies: 14 IKDC: 47.9 IKDC number of studies: 16 KOOS average: N/A KOOS number of studies: N/A Tegner average: N/A Tegner number of studies: N/A Lysholm average: 58.93 Lysholm number of studies: 30	VAS average: 2.31 VAS number of studies: 14 IKDC: 70.06 IKDC number of studies: 16 KOOS average: N/A KOOS number of studies: N/A Tegner Average: N/A Tegner number of studies: N/A Lysholm average: 81.91 Lysholm number of studies: 30	Mean range between 1 and 12.25 years		
Fanelli et al. [16]	Medial MAT	VAS average: N/A VAS number of studies: N/A IKDC: N/A IKDC number of studies: N/A KOOS average: N/A KOOS number of studies: N/A Tegner average: N/A Tegner number of studies: N/A Lysholm average: N/A Lysholm number of studies: N/A	VAS average: 2.6 VAS number of studies: N/A IKDC: 58.25 IKDC number of studies: N/A KOOS average: N/A KOOS number of studies: N/A Tegner average: 5.1 Tegner number of studies: N/A Lysholm average: 75.83 Lysholm number of studies: N/A	5 ± 7 years		Failure rate: 20.48%
Waugh et al. [62]	MAT	VAS average: N/A VAS number of studies: N/A IKDC: 45.33 IKDC number of studies: 9 KOOS average: N/A KOOS number of studies: N/A Tegner average: 3.78 Tegner number of studies: 5 Lysholm average: 50.5 Lysholm number of studies: 10	VAS average: N/A VAS number of studies: N/A IKDC: 70.4 IKDC number of studies: 8 KOOS average: N/A KOOS number of studies: N/A Tegner average: 5.78 Tegner number of studies: 5 Lysholm Average: 74 Lysholm number of studies: 9	2.5 to 17.3 years	Survivorship 1: Percentage: 86.46% Follow‐up: 5 year Survivorship 2: Percentage: 71.73% Follow‐up: 10 year	
Novaretti et al. [39]	MAT	VAS average: N/A VAS number of studies: N/A IKDC: N/A IKDC number of studies: N/A KOOS average: N/A KOOS number of studies: N/A Tegner range: 1–3 Tegner number of studies: 2 Lysholm range: 36–60.5 Lysholm number of studies: 4	VAS average: N/A VAS number of studies: N/A IKDC range: 46‐77 IKDC number of studies: 2 KOOS average: N/A KOOS number of studies: N/A Tegner range: 2.5–4.6 Tegner number of studies: 2 Lysholm range: 61–75 Lysholm number of studies: 4			
Grassi et al. [18]	MAT	VAS average: N/A VAS number of studies: N/A IKDC: 50.9 IKDC number of studies: 5 KOOS average: N/A KOOS number of studies: N/A Tegner average: 2.8 Tegner number of studies: 5 Lysholm average: N/A Lysholm number of studies: N/A	VAS average: N/A VAS number of studies: N/A IKDC: 80.9 IKDC number of studies: 5 KOOS average: N/A KOOS number of studies: N/A Tegner average: 5.2 Tegner number of studies: 5 Lysholm average: N/A Lysholm number of studies: N/A	3.4 ± 1.2 years		Failure rate: 13% Number of studies: 8 Reoperation rate: 23% Number of studies: 8
Lee [30]	MAT	VAS average: N/A VAS number of studies: N/A IKDC: N/A IKDC number of studies: N/A KOOS average: N/A KOOS number of studies: N/A Tegner average: N/A Tegner number of studies: N/A Lysholm average: N/A Lysholm number of studies: N/A	VAS average: N/A VAS number of studies: N/A IKDC: N/A IKDC number of studies: N/A KOOS average: N/A KOOS number of studies: N/A Tegner average: N/A Tegner number of studies: N/A Lysholm average: N/A Lysholm number of studies: N/A	24.9–180.0 months		
Jauregui et al. [23]	MAT	VAS average: 5.6 VAS number of studies: N/A IKDC: N/A IKDC number of studies: N/A KOOS average: N/A KOOS number of studies: N/A Tegner average: N/A Tegner number of studies: N/A Lysholm average: 57.8 Lysholm number of studies: N/A	VAS average: 2.1 VAS number of studies: N/A IKDC: N/A IKDC number of studies: N/A KOOS average: N/A KOOS number of studies: N/A Tegner average: N/A Tegner number of studies: N/A Lysholm average: 81.4 Lysholm number of studies: N/A	61 months		Failure rate: 12.6%
Bin et al. [8]	lateral MAT	VAS average: N/A VAS number of studies: N/A IKDC: N/A IKDC number of studies: N/A KOOS average: N/A KOOS number of studies: N/A Tegner average: N/A Tegner number of studies: N/A Lysholm average: N/A Lysholm number of studies: N/A	VAS average: N/A VAS number of studies: N/A IKDC: N/A IKDC number of studies: N/A KOOS average: N/A KOOS number of studies: N/A Tegner average: N/A Tegner number of studies: N/A Lysholm Average: 72 Lysholm number of studies: 9		Survivorship 1: Percentage: 89.20% Follow‐up: 5–10 year Survivorship 2: Percentage: 56.6% Follow‐up: >10 year	
Barber‐Westin and Noyes [6]	MAT	VAS average: N/A VAS number of studies: N/A IKDC: N/A IKDC number of studies: N/A KOOS average: N/A KOOS number of studies: N/A Tegner average: N/A Tegner number of studies: N/A Lysholm average: N/A Lysholm number of studies: N/A	VAS average: N/A VAS number of studies: N/A IKDC: N/A IKDC number of studies: N/A KOOS average: N/A KOOS number of studies: N/A Tegner average: N/A Tegner number of studies: N/A Lysholm average: N/A Lysholm number of studies: N/A	5.0 ± 3.7 years		Failure rate: 7 to 8% (mean, 3 years postoperative) to 55% (mean, 11.2 years postoperative) Number of studies: 7
DeBruycker et al. [14]	MAT	VAS average: N/A VAS number of studies: N/A IKDC: N/A IKDC number of studies: N/A KOOS average: N/A KOOS number of studies: N/A Tegner average: N/A Tegner number of studies: N/A Lysholm average: N/A Lysholm number of studies: N/A	VAS average change: 4 point decrease VAS number of studies: N/A IKDC average change: 24 point increase IKDC number of studies: N/A KOOS average: N/A KOOS number of studies: N/A Tegner average: N/A Tegner number of studies: N/A Lysholm average change: 20 point increase Lysholm number of studies: N/A	5.4 years	Survivorship 1: Percentage: ~90% Follow‐up: <3 year Survivorship 2: Percentage: 80.9% Follow‐up: >6 year	Failure rate: 19.1% Number of studies: 54
Dangelmajer et al. [13]	MAT	VAS average: N/A VAS number of studies: N/A IKDC: N/A IKDC number of studies: N/A KOOS average: N/A KOOS number of studies: N/A Tegner average: N/A Tegner number of studies: N/A Lysholm average: N/A Lysholm number of studies: N/A	VAS average: N/A VAS number of studies: N/A IKDC: N/A IKDC number of studies: N/A KOOS Average: N/A KOOS number of studies: N/A Tegner average: N/A Tegner number of studies: N/A Lysholm average: N/A Lysholm number of studies: N/A		Survivorship 1: Percentage: 93.5% Follow‐up: 37.2 months Survivorship 2: Percentage: 95% Follow‐up: 5 year	Failure rate: 0%–33.3% (range) Number of studies: 15 Reoperation rate: Range: 0%–33% Number of studies: 10
Wei et al. [63]	Medial MAT	VAS average: N/A VAS number of studies: N/A IKDC: N/A IKDC number of studies: N/A KOOS average: N/A KOOS number of studies: N/A Tegner average: N/A Tegner number of studies: N/A Lysholm average: N/A Lysholm number of studies: N/A	VAS average: N/A VAS number of studies: 5 IKDC: N/A IKDC number of studies: 5 KOOS average: N/A KOOS number of studies: 5 Tegner average: N/A Tegner number of studies: 5 Lysholm average: N/A Lysholm number of studies: 10	Ranged from 24.9 to 165.6 months		Failure rate: 21%
Smith et al. [52]	MAT	VAS average: N/A VAS number of studies: N/A IKDC: 47.8 IKDC number of studies: 12 KOOS average: N/A KOOS number of studies: N/A Tegner average: 3.1 Tegner number of studies: 11 Lysholm average: 55.7 Lysholm number of studies: 25	VAS average: N/A VAS number of studies: N/A IKDC: 70 IKDC number of studies: 12 KOOS average: N/A KOOS number of studies: N/A Tegner average: 4.7 Tegner number of studies: 11 Lysholm average: 81.3 Lysholm number of studies: 25			
Rosso et al. [44]	MAT	VAS average: 6.4 VAS number of studies: N/A IKDC: N/A IKDC number of studies: N/A KOOS average: N/A KOOS number of studies: N/A Tegner average: N/A Tegner number of studies: N/A Lysholm average: 55.5 Lysholm number of studies: N/A	VAS average: 2.4 VAS number of studies: N/A IKDC: N/A IKDC number of studies: N/A KOOS average: N/A KOOS number of studies: N/A Tegner average: N/A Tegner number of studies: N/A Lysholm average: 82.7 Lysholm number of studies: N/A			
Noyes and Barber‐Westion [40]	MAT	VAS average: N/A VAS number of studies: N/A IKDC: N/A IKDC number of studies: N/A KOOS average: N/A KOOS number of studies: N/A Tegner average: N/A Tegner number of studies: N/A Lysholm average: N/A Lysholm number of studies: N/A	VAS average: N/A VAS number of studies: 1 IKDC: N/A IKDC number of studies: 2 KOOS average: N/A KOOS number of studies: 1 Tegner average: N/A Tegner number of studies: 1 Lysholm average: N/A Lysholm number of studies: 7			
Myers and Tudor [38]	MAT	VAS average: N/A VAS number of studies: N/A IKDC: N/A IKDC number of studies: N/A KOOS average: N/A KOOS number of studies: N/A Tegner average: N/A Tegner number of studies: N/A Lysholm average: N/A Lysholm number of studies: N/A	VAS average: N/A VAS number of studies: 12 IKDC: N/A IKDC number of studies: 17 KOOS average: N/A KOOS number of studies: 8 Tegner average: N/A Tegner number of studies: 13 Lysholm average: N/A Lysholm number of studies: 30	4.8 years		
Harris et al. [20]	Combined MAT and cartilage repair/restoration	VAS average: N/A VAS number of studies: N/A IKDC: 32.5 IKDC number of studies: 2 KOOS average: 51 KOOS number of studies: 2 Tegner average: N/A Tegner number of studies: N/A Lysholm average: 47.5 Lysholm number of studies: 4	VAS average: N/A VAS number of studies: N/A IKDC: 65 IKDC number of studies: 2 KOOS average: 82.5 KOOS number of studies: 2 Tegner average: N/A Tegner number of studies: N/A Lysholm average: 73.75 Lysholm number of studies: 4	36 months	Survivorship 1: Percentage: 81% Follow‐up: 10 years Survivorship 2: Percentage: 71% Follow‐up: >10 year	Failure rate: 16% Number of studies: 3
Elattar et al. [15]	MAT	VAS average: 4.8 VAS number of studies: 6 IKDC: N/A IKDC number of studies: N/A KOOS average: N/A KOOS number of studies: 5 Tegner average: 3 Tegner number of studies: 11 Lysholm average: 44 Lysholm number of studies: 24	VAS average: 1.7 VAS number of studies: 6 IKDC: N/A IKDC number of studies: 15 KOOS average: N/A KOOS number of studies: 5 Tegner average: 5 Tegner number of studies: 11 Lysholm average: 77 Lysholm number of studies: 24	4.6 years		Failure rate: 10.6%
Hergan et al. [21]	MAT	VAS average: N/A VAS number of studies: 3 IKDC: N/A IKDC number of studies: 10 KOOS average: N/A KOOS number of studies: 5 Tegner average: N/A Tegner number of studies: 12 Lysholm average: N/A Lysholm number of studies: N/A	VAS average: N/A VAS number of studies: 3 IKDC: N/A IKDC number of studies: 10 KOOS average: N/A KOOS number of studies: 5 Tegner average: N/A Tegner number of studies: 12 Lysholm average: N/A Lysholm number of studies: N/A	53.8 months		Failure rate: 0–37.5% (range) Number of studies: 8
Matava [34]	MAT	VAS average: N/A VAS number of studies: N/A IKDC: N/A IKDC number of studies: N/A KOOS average: N/A KOOS number of studies: N/A Tegner average: N/A Tegner number of studies: N/A Lysholm average: N/A Lysholm number of studies: N/A	VAS average: 1.96 VAS number of studies: 1 IKDC: N/A IKDC number of studies: 3 KOOS average: N/A KOOS number of studies: N/A Tegner average: 4.525 Tegner number of studies: 4 Lysholm average: N/A Lysholm number of studies: 6			

### Return to sport following meniscal allograft transplantation

There were five additional studies that examined return to sport (RTS) after MAT [6, 22, 27, 31, 46]. These findings suggest that while MAT can facilitate RTS for many patients, careful consideration of individual factors is necessary to manage expectations and optimize outcomes. Knapik and colleagues identified factors such as optimal graft sizing, secure fixation, younger age and absence of articular cartilage injury were linked to better RTS outcomes [27]. Conversely, factors like focal chondral defects, osteoarthritic changes, limb malalignment, concomitant procedures during MAT and ligamentous instability were associated with lower RTS success. Hurley and colleagues reviewed 11 studies with 624 patients and found that 77.4% ultimately RTS with 68.6% returning to the same or higher level, at a mean return time of 9.0 months [22]. Lee and colleagues found a similar rate of RTS following MAT with 67% to 85.7% of athletes RTS at an average return time ranging from 7.6 to 16.5 months [31]. Barber‐Westin and colleagues, however, concluded that although the majority of individuals returned to low‐impact athletic activities after MAT, they did not have enough evidence to make a conclusion about high‐impact activities [6]. Similarly, Samitier and colleagues found that the majority of individuals returned to low‐impact athletic activities after meniscus transplantation [46]. They were able to conclude, however, that MAT enables a RTS at the same level of competition in 75%–85% of patients at short‐ to mid‐term follow‐up.

### Fixation of meniscal allograft transplant

Several studies compared MAT root configuration technique by examining differences in PROMs and outcomes between soft tissue fixation compared to bone fixation. Seitz and colleagues conducted a review on biomechanical studies and found that attaching allografts using bone plugs or a bone block was more effective than circumferential suturing when evaluating the chondroprotective biomechanical function [48]. Samitier and colleagues reported that bone fixation was superior in restoring contact mechanics compared to suture fixation, while suture fixation carried a higher risk of graft extrusion, but ultimately found no differences in pullout strength or functional outcomes [45]. A review comparing clinical outcomes between soft tissue suture (485 MATs) and bone fixation (489 MATs) groups found no significant differences in allograft tear rates (13.4% vs. 14.9%), failure rates (17.6% vs. 18.8%) and PROMS (Lysholm and VAS scores) [23]. Similarly, Myers and colleagues found that the evidence supporting fixation with either bone plugs or soft tissue was inconclusive while Leite and colleagues reported a similar rate of allograft extrusion between the two techniques [32, 38]. However, Fanelli and colleagues found that soft tissue fixation resulted in superior postoperative IKDC scores compared to bone fixation. A meta‐analysis by Ow and colleagues directly compared bone plug fixation compared to suture fixation and bone bridge methods [42]. They found that utilizing bone plug fixation for MAT was associated with a reduced risk of failure compared to the bone bridge method. Moreover, it demonstrated a lower likelihood of requiring further operations than both suture‐only and bone bridge fixation methods.

## DISCUSSION

The purpose of this umbrella review, focusing on systematic reviews and meta‐analyses of meniscal transplantation, was to identify literature gaps and propose directions for future research. Part two of the umbrella review focuses on scaffold‐based approaches for meniscal deficiency [64]. The current literature base has consistently demonstrated favourable short‐term outcomes when it comes to PROMs in MAT, but the reviews are limited by the lack of randomized control trials and consistent comparison groups. Multiple reviews have notably highlighted the challenges associated with drawing strong conclusions. These challenges arise from the differences in methodologies among the included studies, including variation in concomitant procedures.

Our review highlights an increased interest in MAT, particularly evident in the substantial number of reviews published within the last decade. However, the quality of these reviews has been a limiting factor, with many lacking standardized reporting and rigorous methodologies. The methodological quality assessment of the included studies revealed significant weaknesses, with most studies receiving low to critically low ratings on the AMSTAR‐2 criteria. Common issues included lack of protocol registration, inadequate risk of bias assessment, and failure to address conflicts of interest. These shortcomings raise concerns about the reliability and validity of systematic and meta‐analysis reviews on MAT. These weaknesses have implications for clinical practice and patient outcomes. The questionable reliability of current evidence makes it challenging for clinicians to confidently utilize these findings to guide patient care. Inappropriate treatment decisions based on unreliable evidence could lead to suboptimal outcomes and patient dissatisfaction. Improving the quality of future research in this area necessitates standardizing outcome measures and protocols for assessing risk of bias to enhance study comparability and reliability. Additionally, promoting transparency and rigor in reporting methods and results is crucial to addressing these identified weaknesses. Collaborative efforts among researchers, clinicians and journal editors may be necessary to effectively establish and enforce these standards. Future reviews could benefit from a more comprehensive approach, including a thorough assessment of risk of bias, adherence to PRISMA guidelines and consideration of the risk of bias assessment during evidence synthesis.

Additionally, there is a need for reviews to address key gaps in our understanding, such as the optimal surgical techniques, and patient selection criteria. The literature on long‐term outcomes of MAT highlights the importance of considering the possibility of conversion to total knee arthroplasty (TKA) post‐MAT. Several studies and reviews have highlighted the need for conversion in the 10 years following MAT [15, 25, 28, 39]. Clinicians need to weigh the potential benefits of MAT against the risk of future TKA when discussing treatment options with patients. Research focused on factors influencing the likelihood of TKA after MAT, such as patient age, activity level, degree of cartilage damage and knee joint alignment, can provide clinicians with valuable insights to counsel patients effectively about the risks and lifestyle considerations associated with MAT.

Although there have been reviews focused on surgical techniques within MAT, there has been a lack of consensus for best practices. Furthermore, efforts to standardize reporting and outcome measures across studies would greatly enhance the comparability and reliability of findings in this field. Multiple reviews have highlighted that the absence of a uniform definition of failure and inconsistent use of outcome scores have restricted the conclusiveness of findings [38]. The reviews discussing fixation techniques were hindered by a generally low quality of evidence and considerable heterogeneity among the studies included [32].

There is a lack of information regarding the use of MAT in pediatric patients [56]. Only one review examined the use of MAT exclusively in skeletally immature patients and found that it was safe with positive short‐term clinical outcomes [57]. However, the study was limited by the lack of long‐term data and the inclusion of only 3 articles with 58 total patients. The increasing participation of children in sports, including at elite levels, has led to a higher incidence of sports‐related injuries in this population [4, 58]. Consequently, more children are experiencing early meniscus loss, which can contribute to progressive degenerative joint disease. The limited number of reviews underscores the lack of high‐quality studies focusing on indications, timing and outcomes of MAT in pediatric populations.

Our review builds upon the work of Smoak and colleagues, who conducted a systematic review of the meniscus literature [53]. However, unlike their study, we have rigorously assessed the quality of each review and identified potential areas for improvement. This critical appraisal of the literature distinguishes our review as the first to systematically evaluate and appraise each article, providing a more comprehensive understanding of the current state of research in this field.

One limitation of this umbrella review is its restriction to studies published in English and indexed in the MEDLINE, Embase and Scopus databases, potentially overlooking relevant publications in other languages. Another limitation is the inclusion of all systematic reviews regardless of their methodological quality, could affect the validity of the review and the conclusions. It is important to note that flaws in critical domains, which significantly influence the final overall quality rating of a review, could impact the reliability of the findings. Furthermore, the heterogeneity among the included reviews in terms of patient populations, surgical techniques and outcome measures may introduce variability in the conclusion and limit the generalizability of the findings. Differences in reporting standards across reviews may hinder the ability to compare results and draw definitive conclusions. Additionally, interpreting findings from multiple reviews with different methodologies and outcomes is complex and can lead to misinterpretation.

## CONCLUSION

This umbrella review provides a comprehensive assessment of the current state of research on MAT through a synthesis of 41 systematic reviews and meta‐analyses. While the literature demonstrates favourable short‐term outcomes in terms of PROMs following MAT, the overall methodological quality of the included studies is concerning, with most reviews receiving low to critically low ratings on the AMSTAR‐2 criteria. These findings highlight the need for higher‐quality reviews with standardized reporting and rigorous methodologies. Future research should focus on optimal surgical techniques, patient selection criteria and risk factors for transplant failure. Additionally, there is a need for more studies focusing on MAT in pediatric populations. Overall, this review underscores the importance of critically evaluating the existing evidence base to guide clinical practice and improve patient outcomes in MAT.

## AUTHOR CONTRIBUTIONS

All authors contributed to the idea, initiation, execution and revision of the study. Kevin A. Wu wrote the original draft. Stephanie Hendren performed the literature search. Lulla V. Kiwinda, Aaron D. Therien and Christian J. Castillo collated data. Kevin A. Wu, Lulla V. Kiwinda, Aaron D. Therien analyzed the data. Jason S. Long, Annunziato Amendola, Brian C. Lau conceptualized the project and gave critical feedback. All authors agreed on the order of authorship prior to manuscript submission. The authors read and approved the final manuscript.

## CONFLICT OF INTEREST STATEMENT

The authors declare no conflict of interest.

## ETHICS STATEMENT

This is a review study. The Duke Health Research Ethics Committee has confirmed that no ethical approval is required.

## Supporting information

Supporting information.

## Data Availability

Data sharing is not applicable to this article as no new data were created or analyzed in this study.
