# Supplementary material for: Addressing meniscal deficiency part 1: An umbrella review of systematic reviews and meta‐analyses on meniscal allograft transplantation
Source: J Exp Orthop. 2024 Sep 30;11(4):e12107. doi: 10.1002/jeo2.12107 (PMC11440370; doi:10.1002/jeo2.12107)
Supplement: Supplementary file 1 — Supporting information. [file JEO2-11-e12107-s001.docx]

**Appendix. Search Strategies**

Steph Hendren, MLIS; Duke University Medical Center Library, Duke University School of Medicine

Date of Original Search: September 9, 2021

Date of Updated Search: October 23, 2023

Database: MEDLINE (PubMed)

| Search Concepts | Search Strings | Original Search (9.9.2021) | Updated Search (10.23.2023) |
| --- | --- | --- | --- |
| 1.  Meniscus keyterms | "Meniscus"[Mesh] OR "Menisci, Tibial"[Mesh] OR "Tibial Meniscus Injuries"[Mesh] OR "Meniscectomy"[Mesh] OR meniscal[tiab] OR meniscus[tiab] OR menisci[tiab] OR meniscectomy[tiab] OR meniscectomies[tiab] OR "Semilunar Cartilage"[tiab] OR "Semilunar Cartilages"[tiab] | 19721 | 22558 |
| 2.  scaffold/transplant keyterms | "Transplantation"[Mesh] OR "Transplants"[Mesh] OR "Allografts"[Mesh] OR "Collagen"[Mesh] OR "Polyurethanes"[Mesh] OR "Tissue Scaffolds"[Mesh] OR implant[tiab] OR implants[tiab] OR implantation[tiab] OR implantations[tiab] OR implanting[tiab] OR implanted[tiab] OR scaffold[tiab] OR scaffolds[tiab] OR scaffolding[tiab] OR scaffoldings[tiab] OR transplant[tiab] OR transplants[tiab] OR transplantation[tiab] OR transplantations[tiab] OR transplanting[tiab] OR transplanted[tiab] OR allotransplant[tiab] OR allotransplantation[tiab] OR allograft[tiab] OR allografts[tiab] OR allografting[tiab] OR MAT[tiab] OR MATs[tiab] OR CMI[tiab] OR CMIs[tiab] OR actifit[tiab] OR menaflex[tiab] OR polycaprolactone–polyurethane[tiab] OR polyurethane[tiab] OR polyurethanes[tiab] OR collagen[tiab] | 1435066 | 1588955 |
| 3. systematic review filter (modified from the CADTH filter)** | "systematic"[filter] OR "meta-analysis"[pt] OR "meta-analysis as topic"[mh] OR "Clinical protocols"[MESH] OR "Consensus"[MESH] OR "Critical pathways"[MESH] OR "Guidelines as topic" OR "Practice guidelines as topic"[MESH] OR "Health planning guidelines"[MESH] OR "Clinical Decision Rules"[MESH] OR meta analy*[tw] OR metanaly*[tw] OR metaanaly*[tw] OR met analy*[tw] OR integrative research[tiab]  OR integrative review*[tiab] OR integrative overview*[tiab] OR research integration*[tiab] OR research overview*[tiab] OR collaborative review*[tiab] OR collaborative overview*[tiab] OR "systematic review"[pt] OR "systematic reviews as topic"[mh] OR systematic review*[tiab] OR Embase*[tiab] OR Cinahl*[tiab] OR systematic overview*[tiab] OR methodological overview*[tiab]  OR methodologic overview*[tiab]  OR methodological review*[tiab]  OR methodologic review*[tiab] OR quantitative review*[tiab] OR  quantitative overview*[tiab] OR quantitative synthes*[tiab] OR pooled analy*[tiab] OR Cochrane[tiab] OR Medline[tiab] OR Pubmed[tiab] OR Medlars[tiab] OR handsearch*[tiab] OR hand search*[tiab] OR meta-regression*[tiab] OR metaregression*[tiab] OR data synthes*[tiab] OR data extraction[tiab] OR data abstraction*[tiab] OR mantel haenszel[tiab] OR peto[tiab] OR der-simonian[tiab] OR dersimonian[tiab] OR fixed effect*[tiab] OR multiple treatment comparison[tiab] OR mixed treatment meta-analys*[tiab] OR umbrella review*[tiab] OR ((multiple paramet*[tiab]) AND (evidence synthesis[tiab]))  OR ((multi-paramet*[tiab]) AND (evidence synthesis[tiab])) OR ((multiparameter*[tiab]) AND (evidence synthesis[tiab])) OR "guideline"[pt] OR "practice guideline"[pt] OR "consensus development conference"[pt] OR "consensus development conference, NIH"[pt] OR position statement*[tiab] OR policy statement*[tiab] OR practice parameter*[tiab] OR best practice*[tiab] OR standards[TI] OR guideline[TI] OR guidelines[TI] OR standards[ot] OR guideline[ot] OR guidelines[ot] OR guideline*[cn] OR standards[cn] OR consensus*[cn] OR recommendat*[cn] OR practice guideline*[tiab] OR treatment guideline*[tiab] OR CPG[tiab] OR CPGs[tiab] OR clinical guideline*[tiab] OR guideline recommendation*[tiab] OR consensus*[tiab] OR ((critical[tiab] OR clinical[tiab] OR practice[tiab]) AND (path[tiab] OR paths[tiab] OR pathway[tiab] OR pathways[tiab] OR protocol*[tiab] OR bulletin[tiab] OR bulletins[tiab])) OR recommendat*[ti] OR recommendat*[ot] OR (care[tiab] AND (standard[tiab] OR path[tiab] OR paths[tiab] OR pathway[tiab] OR pathways[tiab] OR map[tiab] OR maps[tiab] OR plan[tiab] OR plans[tiab])) OR (algorithm*[tiab] AND (screening[tiab] OR examination[tiab] OR test[tiab] OR tested[tiab] OR testing[tiab] OR assessment*[tiab] OR diagnosis[tiab] OR diagnoses[tiab] OR diagnosed[tiab] OR diagnosing[tiab])) OR (algorithm*[tiab] AND (pharmacotherap*[tiab] OR chemotherap*[tiab] OR chemotreatment*[tiab] OR therap*[tiab] OR treatment*[tiab] OR intervention*[tiab])) | 1771392 | 2142011 |
| 4. | 1 AND 2 AND 3 | 227 | 284 |

**Modified CADTH filters for systematic reviews and guidelines (removed the keyterms for health technology assessment, the specific journal titles)

Database: Embase (Elsevier)

Note: all searches conducted on "results" page

| Search Concepts | Search Strings | Original Search (9.9.2021) | Updated Search (10.23.2023) |
| --- | --- | --- | --- |
| 1.  Meniscus keyterms | 'knee meniscus'/exp OR 'knee meniscus rupture'/exp OR 'meniscectomy'/exp OR (meniscal OR meniscus OR menisci OR meniscectomy OR meniscectomies OR 'Semilunar Cartilage' OR 'Semilunar Cartilages'):ti,ab | 28703 | 33547 |
| 2.  scaffold/transplant keyterms | 'transplantation'/exp OR 'allograft'/exp OR 'collagen'/exp OR 'polyurethan'/exp OR 'tissue scaffold'/exp OR (implant OR implants OR implantation OR implantations OR implanting OR implanted OR scaffold OR scaffolds OR scaffolding OR scaffoldings OR transplant OR transplants OR transplantation OR transplantations OR transplanting OR transplanted OR allotransplant OR allotransplantation OR allograft OR allografts OR allografting OR MAT OR MATs OR CMI OR CMIs OR actifit OR menaflex OR polycaprolactone–polyurethane OR polyurethane OR polyurethanes OR collagen):ti,ab | 2240147 | 2516429 |
| 3. systematic review filter (modified from the CADTH filter)** | 'systematic review'/exp OR 'systematic review (topic)'/exp OR 'meta analysis (topic)'/exp OR 'meta analysis'/exp OR 'clinical protocol'/exp OR 'consensus'/exp OR 'clinical pathway'/exp OR 'practice guideline'/exp OR 'practice guideline'/exp OR 'health care planning'/exp OR 'clinical decision rule'/exp OR ('meta analy*' OR metanaly* OR metaanaly* OR 'met analy*' OR 'integrative research' OR 'integrative review*' OR 'integrative overview*' OR 'research integration*' OR 'research overview*' OR 'collaborative review*' OR 'collaborative overview*' OR 'systematic review*' OR Embase* OR Cinahl* OR 'systematic overview*' OR 'methodological overview*' OR 'methodologic overview*' OR 'methodological review*' OR 'methodologic review*' OR 'quantitative review*' OR 'quantitative overview*' OR 'quantitative synthes*' OR 'pooled analy*' OR Cochrane OR Medline OR Pubmed OR Medlars OR handsearch* OR 'hand search*' OR meta-regression* OR metaregression* OR 'data synthes*' OR 'data extraction' OR 'data abstraction*' OR 'mantel haenszel' OR peto OR der-simonian OR dersimonian OR 'fixed effect*' OR 'multiple treatment comparison' OR 'mixed treatment meta-analys*' OR 'umbrella review*'):ti,ab OR (('multiple paramet*' OR multi-paramet OR multiparameter*) AND ('evidence synthesis')):ti,ab OR ('position statement*' OR 'policy statement*' OR 'practice parameter*' OR 'best practice*' OR guideline OR guidelines OR consensus OR recommendat*):ti,ab OR standards:ti OR ('practice guideline*' OR 'treatment guideline*' OR CPG OR CPGs OR 'clinical guideline*' OR 'guideline recommendation*' OR consensus*):ti,ab OR ((critical OR clinical OR practice) AND (path OR paths OR pathway OR pathways OR protocol* OR bulletin OR bulletins)):ti,ab OR recommendat*:ti,ab OR (care AND (standard OR path OR paths OR pathway OR pathways OR map OR maps OR plan OR plans)):ti,ab OR (algorithm* AND (screening OR examination OR test OR tested OR testing OR assessment* OR diagnosis OR diagnoses OR diagnosed OR diagnosing)):ti,ab OR (algorithm* AND (pharmacotherap* OR chemotherap* OR chemotreatment* OR therap* OR treatment* OR intervention*)):ti,ab | 3061075 | 3699965 |
| 4. | 1 AND 2 AND 3 | 614 | 818 |

Database: Scopus (Elsevier)

| Search Concepts | Search Strings | Original Search (9.9.2021) | Updated Search (10.23.2023) |
| --- | --- | --- | --- |
| 1.  Meniscus keyterms | TITLE-ABS(meniscal OR meniscus OR menisci OR meniscectomy OR meniscectomies OR {Semilunar Cartilage} OR {Semilunar Cartilages}) | 29642 | 33704 |
| 2.  scaffold/transplant keyterms | TITLE-ABS(implant OR implants OR implantation OR implantations OR implanting OR implanted OR scaffold OR scaffolds OR scaffolding OR scaffoldings OR transplant OR transplants OR transplantation OR transplantations OR transplanting OR transplanted OR allotransplant OR allotransplantation OR allograft OR allografts OR allografting OR MAT OR MATs OR CMI OR CMIs OR actifit OR menaflex OR polycaprolactone–polyurethane OR polyurethane OR polyurethanes OR collagen) | 1640067 | 1845412 |
| 3. systematic review filter (modified from the CADTH filter)** | TITLE-ABS(systematic OR "meta analy*" OR metanaly* OR metaanaly* OR "met analy*" OR "integrative research" OR "integrative review*" OR "integrative overview*" OR "research integration*" OR "research overview*" OR "collaborative review*" OR "collaborative overview*" OR Embase* OR Cinahl* OR "systematic overview*" OR "methodological overview*" OR "methodologic overview*" OR "methodological review*" OR "methodologic review*" OR "quantitative review*" OR "quantitative overview*" OR "quantitative synthes*" OR "pooled analy*" OR Cochrane OR Medline OR Pubmed OR Medlars OR handsearch* OR "hand search*" OR meta-regression* OR metaregression* OR "data synthes*" OR "data extraction" OR "data abstraction*" OR "mantel haenszel" OR peto OR der-simonian OR dersimonian OR "fixed effect*" OR "multiple treatment comparison" OR "umbrella review*") OR TITLE-ABS((("multiple paramet*") AND ("evidence synthesis"))  OR ((multi-paramet*) AND ("evidence synthesis")) OR ((multiparameter*) AND ("evidence synthesis"))) OR TITLE(guideline OR guidelines OR standard OR standards OR recommendation OR recommendations) OR TITLE-ABS("position statement*" OR "policy statement*" OR "practice parameter*" OR "best practice*" OR "practice guideline*" OR "treatment guideline*" OR CPG OR CPGs OR "clinical guideline*" OR "guideline recommendation*" OR consensus*) OR TITLE-ABS(((critical OR clinical OR practice) AND (path OR paths OR pathway OR pathways OR protocol* OR bulletin OR bulletins)) OR recommendat* OR recommendat* OR (care AND (standard OR path OR paths OR pathway OR pathways OR map OR maps OR plan OR plans)) OR (algorithm* AND (screening OR examination OR test OR tested OR testing OR assessment* OR diagnosis OR diagnoses OR diagnosed OR diagnosing)) OR (algorithm* AND (pharmacotherap* OR chemotherap* OR chemotreatment* OR therap* OR treatment* OR intervention*))) | 3723230 | 4544099 |
| 4. | 1 AND 2 AND 3 | 250 | 320 |
